# The monocyte-to-high-density lipoprotein ratio is associated with the occurrence of atrial fibrillation among NAFLD patients: A propensity-matched analysis

**DOI:** 10.3389/fendo.2023.1127425

**Published:** 2023-03-28

**Authors:** Leigang Wang, Yao Zhang, Bing Yu, Jianqi Zhao, Wenjing Zhang, Hongxuan Fan, Zhaoyu Ren, Bin Liang

**Affiliations:** ^1^ Shanxi Medical University, Taiyuan, Shanxi, China; ^2^ Department of Cardiovascular Medicine, Second Hospital of Shanxi Medical University, Taiyuan, Shanxi, China

**Keywords:** monocyte to high-density lipoprotein cholesterol ratio, inflammation, atrial fibrillation, nonalcoholic fatty liver disease, propensity score matching analysis

## Abstract

**Background:**

Accumulating evidence suggests that patients with nonalcoholic fatty liver disease (NAFLD) have a significantly high risk of incident atrial fibrillation (AF). Systemic inflammation, metabolic disorders and oxidative stress could be the potential mechanisms by which NAFLD drives AF. Monocyte-to- high-density lipoprotein ratio (MHR) has emerged as a novel biomarker of inflammation and oxidative stress that has not been studied in AF with NAFLD patients. We aimed to investigate the relationship between MHR and the risk of AF among NAFLD patients.

**Methods:**

A retrospective analysis was performed for the clinical data of the patients with NAFLD in the Second Hospital of Shanxi Medical University from January 2019 to October 2022, among whom 204 patients with AF were enrolled as NAFLD+AF group and 613 patients without AF were enrolled as NAFLD control, and 152 patients were selected from each group based on propensity score matching (PSM) at a ratio of 1:1 to balance the covariates between groups. The t-test or the Mann-Whitney U test was used for comparison of continuous data between two groups; the chi-square test or the Fisher’s exact test was used for comparison of categorical data between two groups. Logistic regression analysis was performed to identify the independent predictor for occurrence of AF among NAFLD patients. Trend chi-square test to analyze the prevalence of AF among MHR tertiles, and then the correlation between MHR and the risk of AF confirmed by restricted cubic splines (RCS). The receiver operating characteristic (ROC) curve analysis was used to determine the optimum MHR cutoff value to predict AF.

**Results:**

Univariate analysis showed that AF patients had higher MHR than non-AF patients (P < 0.001). Meanwhile, compared with pure NAFLD patients, multivariate logistic regression analysis showed that MHR remained to be an independent risk factor for AF after adjusting for confounding risk factors (OR = 10.67, 95% CI 2.17-52.37, P = 0.004). TC、HDL-C were also independent risk factors for AF. Among them, TC and HDL-C are protective factors for AF. The trend chi-square test showed that the risk of AF increased with an increase in MHR (P < 0.05). However, the RCS showed a nonlinear and J-shaped relationship between MHR and the risk of AF (P for non-linearity = 0.023). The occurrence of AF increased with increasing MHR only when MHR > 0.44. The ROC curve showed that MHR combined with traditional risk factors can improve the ability to predict AF.

**Conclusion:**

MHR is an independently associated with incident AF in patients with NAFLD and show a certain predictive value.

## Introduction

Atrial fibrillation (AF) is the most prevalent cardiac arrhythmia in the general population, carrying a considerable risk of morbidity and mortality (1). According to the 2010 Global Burden of Disease Study, the age-adjusted prevalence of AF is 5.96 per 1000 in men and 3.73 per 1000 in women worldwide, resulting in an estimated 33 million persons (2). In view of the emergence of AF as a growing epidemic, the global burden of AF deserves attention. However, to date, there are no reliable biomarkers that can be used to identify patients with asymptomatic or occult paroxysmal AF. Therefore, the search for a novel biomarker to predict the potential of AF is highly warranted, especially in high-risk patients.

Non-alcoholic fatty liver disease (NAFLD) is a common cause of chronic liver disease, and its incidence continues to increase globally (3). And it is characterized by hepatic fat accumulation and hepatic steatosis. NAFLD impacts around 30% of the adult population in many western countries, and its incidence furtherly rises to 70–90% among those with obesity or diabetes, the 2 well-known risk factors for AF (4). Recently, many clinical studies have discovered a significant increase in the number of AF in patients with NAFLD, indicating that NAFLD is an independent risk factor for AF (5–7). As a multisystem disease, NAFLD not only causes liver lesions, but also induces systemic inflammation, oxidative stress and metabolic disorders, which may lead to structural, functional and electrical remodeling of the heart, thereby increasing susceptibility to arrhythmia (8).

The monocyte to high density-lipoprotein (HDL) cholesterol ratio (MHR), a novel indicator of inflammation, oxidative stress and metabolic syndrome, has been proved as a predictor and prognostic marker of cardiovascular diseases (CVD) (9). Previous research has established that high preoperative MHR is associated with postoperative AF and mortality in coronary artery bypass grafting (10). In addition, a prospective and observational study has revealed a correlation between elevated pre-ablation MHR and recurrence of AF after cryoballoon-based catheter ablation (11). Meanwhile, as mentioned in the Cross-Sectional Study, MHR is significantly and positively associated with the risk of NAFLD (12). However, no data was found on the association between MHR and the risk of AF among NAFLD patients. Therefore, this thesis intends to determine the correlation between MHR and the occurrence of AF, and to further investigate whether MHR could become an independent marker to predict AF presence among NAFLD patients.

## Methods

### Study population

We retrospectively analyzed the clinical data of patients who were diagnosed with NAFLD and recorded the surface electrocardiogram (ECG) at the Second Affiliated Hospital of Shanxi Medical University from January 2019 to October 2022. All participants were divided into NAFLD + AF and NAFLD groups based on the current or past ECG. Among them, 94 patients have paroxysmal AF and 58 patients have non-paroxysmal AF.

### Definition and measurement of NAFLD and other diseases

NAFLD was diagnosed (13) based on the criteria recommended by the Chinese Society of Liver Diseases, ultrasound findings of fatty liver, and exclusion of other causes of chronic liver disease. Fatty liver disease was diagnosed in patients with at least two of the following three findings (1): a diffuse enhancement of the liver near-field echo that was stronger than that of the kidney (2), a poorly delineated structure of the intrahepatic bile duct, and (3) a gradual attenuation of the far-field echo of the liver.

According to 2020 European Society of Cardiology guidelines, a standard 12-lead ECG recording or a single-lead ECG tracing of ≥30 s showing heart rhythm with no discernible repeating P waves and irregular RR intervals (when atrioventricular conduction is not impaired) is diagnostic of clinical AF (14). CHD is defined as having at least one coronary artery or its major branches narrowed by > 50% on coronary angiography.

Our exclusion criteria included rheumatic heart disease, structural cardiomyopathy, congenital heart disease, valvular disease, severe heart failure, infectious disease, autoimmune disease, hematologic disease, malignancy, hyperthyroidism, severe hepatorenal insufficiency and a history of chronic alcohol consumption.

This study was approved by the local ethics committee of the Second Affiliated Hospital of Shanxi Medical University. We obtained informed consent from all participants.

### Data collection and measurements

The following information was collected through the electronic medical record system (1): Demographic characteristics: age and gender (2); Previous history: smoking history, hypertension, diabetes and coronary heart disease (CHD) (3); Laboratory indicators: blood count levels (including white blood cells, monocytes, lymphocytes, and neutrophils), total cholesterol (TC), triglycerides (TG), high-density lipoprotein cholesterol (HDL-C) and low density lipoprotein cholesterol (LDL-C), alanine aminotransferase (ALT), aspartate aminotransferase (AST), serum creatinine (Scr) and fasting plasma glucose (FPG). Blood samples were obtained from the patients in the morning after 12 h of fasting. BMI was calculated as the patient’s weight (kg)/height^2^ (m^2^). MHR was calculated as the monocytes count (10^9^/L)/HDL-C (mmol/L), NLR was calculated as the neutrophils count (10^9^/L)/lymphocytes count (10^9^/L), and NHR was calculated as the neutrophils count (10^9^/L)/HDL-C (mmol/L).

### Statistical analysis

The continuous variables were expressed as mean ± standard deviation (SD) or median (IQR) and were compared between groups of patients with or without AF. Categorical variables are expressed as percentages. For univariate analysis between AF and non-AF, the independent samples t-test or Mann-Whitney test was used for comparison of continuous variables, and the chi-square test or Fisher’s exact test was used for comparison between categorical variables. Binary logistic regression analysis was performed to identify independent factors associated with AF among NAFLD, and variables with p-values ​​less than 0.05 in the univariate logistic regression analysis were included in the multivariate logistic regression analysis. chi-square test for trend analysis between multiple groups of categorical variables, Kruskal–Wallis test analysis between multiple groups of continuous variables. We also used restricted cubic splines (RCS) to flexibly model the association of MHR with the prevalent AF. P<0.05 (two-sided) was considered statistically significant. Statistical analysis was carried out using SPSS version 26.0 (IBM SPSS Statistics for Windows, USA) and R version 4.0.1 software.

### Propensity score matching analysis

Propensity score matching (PSM) was utilized to eliminate bias and control for potential confounding variables. A logistic regression model was built with the presence or non-presence of atrial fibrillation as the dependent variable and confounding factors affecting the incidence of atrial fibrillation as the independent variable, including age, gender, BMI, hypertension, diabetes, CHD and smoking history. The propensity score value of each study object was calculated, the calliper value was 0.02. The ability of the matching to balance baseline characteristics in AF *vs*. non-AF was assessed using absolute standard differences and a quartile, reporting a non-significant value of <10%. After PSM (1:1), the AF and non-AF groups were matched, with a good balance between groups.

## Results

### Clinical characteristics of the participants

**Figure 1:**
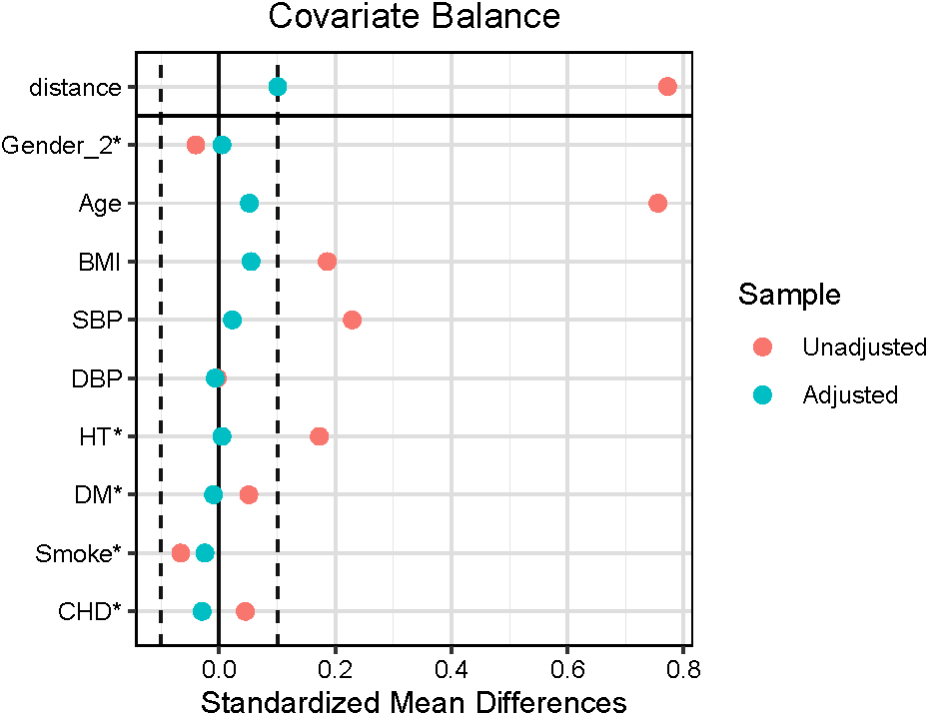
Love plots for absolute standardized differences for baseline covariates of patients between AF and non-AF group, before and after propensity score matching.

**Table 1** shows the characteristics of the patients with AF and controls before and after PSM. After matching, absolute standardized differences for all measured covariates were <10%, indicating significant covariate balance between the two groups (**Figure 1**). Age, gender, CVD, hypertension, DM and stroke did not significantly differ between the groups (P > 0.05) after PSM (**Table 1**).

**Table 1:** Baseline characteristics of patients with NAFLD (control) and with NAFLD and AF.

	Before PSM			After PSM (1:1)		
	AF (204)	non-AF (613)	P	AF (152)	non-AF (152)	P
Gender(male)	132 (64.7)	372 (60.7)	0.306	95 (62.5)	93 (61.2)	0.813
Age(year)	68.83 ± 11.00	56.31 ± 10.39	<0.001	65.05 ± 9.29	63.99 ± 9.29	0.321
BMI(kg/m^2)^	26.67 (24.51,28.58)	25.44 (24.26,27.36)	0.109	25.95 (24.18,27.78)	25.88 (24.27,28.20)	0.215
SBP(mmHg)	136.46 ± 20.49	131.71 ± 18.43	0.002	135.68 ± 19.66	135.82 ± 19.75	0.951
DBP(mmHg)	80.09 ± 14.13	80.13 ± 12.73	0.966	80.53 ± 14.16	80.03 ± 13.39	0.754
HT [(n%)]	149 (73.0)	342 (55.8)	<0.001	110 (72.4)	106 (69.7)	0.613
DM [(n%)]	64 (31.4)	161 (26.3)	0.157	46 (30.3)	47 (30.9)	0.901
CHD[(n%)]	121 (59.3)	336 (54.8)	0.262	95 (62.5)	85 (55.9)	0.243
Smoke[(n%)]	76 (37.3)	269 (43.9)	0.097	59 (38.8)	62 (40.8)	0.725

Data are shown as means ± SD or medians with interquartile ranges (IQRs). AF, atrial fibrillation; BMI, body mass index; SBP, systolic blood pressure; DBP, diastolic blood pressure; HT, hypertension; DM, diabetes mellitus; CHD, Coronary heart disease; PSM, propensity score matching.

**Figure 2:**
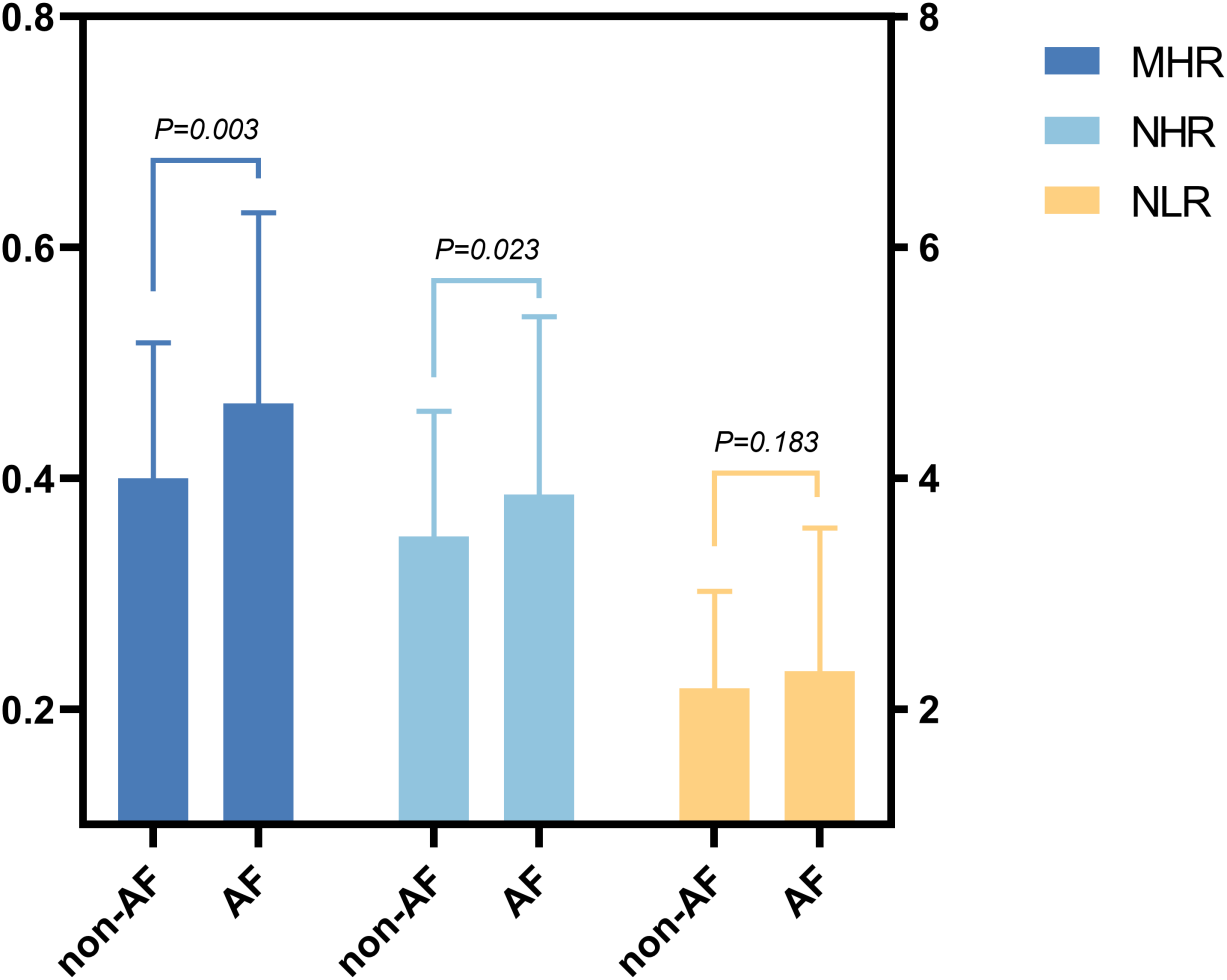
Inflammatory characteristics of patients in AF and non-AF groups.

Meanwhile, the ALT, AST, Scr, TC, TG, LDL-C and HDL-C were significantly different among all groups (P < 0.05; **Table 2**). The inflammatory markers, WBC, MON, NEU, LEM and NLR, did not significantly differ between the two groups (P > 0.05), but levels of MHR and NHR were significantly higher in the AF group (**Table 2**; **Figure 2**).

**Table 2:** Laboratory indicators in patients with AF and non-AF.

	AF (152)	non-AF (152)	P
ALT(U/L)	21.10 (15.13,33.30)	16.20 (13.60,21.28)	<0.001
AST(U/L)	22.45 (18.73,27.60)	16.90 (15.35,21.08)	<0.001
Scr(mmol/L)	70.00 (61.00,80.00)	67.00 (58.00,75.00)	0.003
TC (mmol/L)	3.70 (2.98,4.50)	4.32 (3.53,5.00)	<0.001
TG (mmol/L)	1.41 (1.01,2.06)	1.57 (1.23,2.14)	0.029
HDL-C(mmol/L)	1.02 (0.88,1.20)	1.13 (0.97,1.34)	<0.001
LDL-C(mmol/L)	2.04 (1.48,2.65)	2.24 (1.77,2.70)	0.017
FPG (mmol/L)	5.73 (5.21,7.11)	5.71 (5.07,7.24)	0.314
WBC (10^9^/L)	6.54 (5.50,7.74)	6.64 (5.36,7.69)	0.935
MON (10^9^/L)	0.48 (0.36,0.61)	0.47 (0.37,0.55)	0.341
NEU (10^9^/L)	3.95 (3.25,5.09)	3.84 (3.02,4.99)	0.458
LEM (10^9^/L)	1.66 (1.26,2.11)	1.77 (1.42,2.20)	0.177
MHR	0.47 (0.33,0.63)	0.40 (0.32,0.51)	0.003
NHR	3.86 (2.92,5.40)	3.50 (2.62,4.58)	0.023
NLR	2.33 (1.71,3.57)	2.18 (1.64,3.02)	0.183

Values are expressed as median (interquartile range). P < 0.05 (two-sided) was deﬁned as statistically signiﬁcant.

AF, Atrial fibrillation; ALT, alanine aminotransferase; AST, aspartate aminotransferase; Scr, serum creatinine; TC, total cholesterol; TG, triglycerides; HDL-C, high-density lipoprotein cholesterol; LDL-C, low-density lipoprotein cholesterol; FPG, fasting plasma glucose; WBC, White blood cell; MHR, monocyte to high-density lipoprotein cholesterol ratio; NHR, neutrophil to high-density lipoprotein cholesterol ratio; NLR, neutrophil to lymphocyte ratio.

### Univariate and multivariate analyses of factors associated with AF in NAFLD

**Figure 3:**
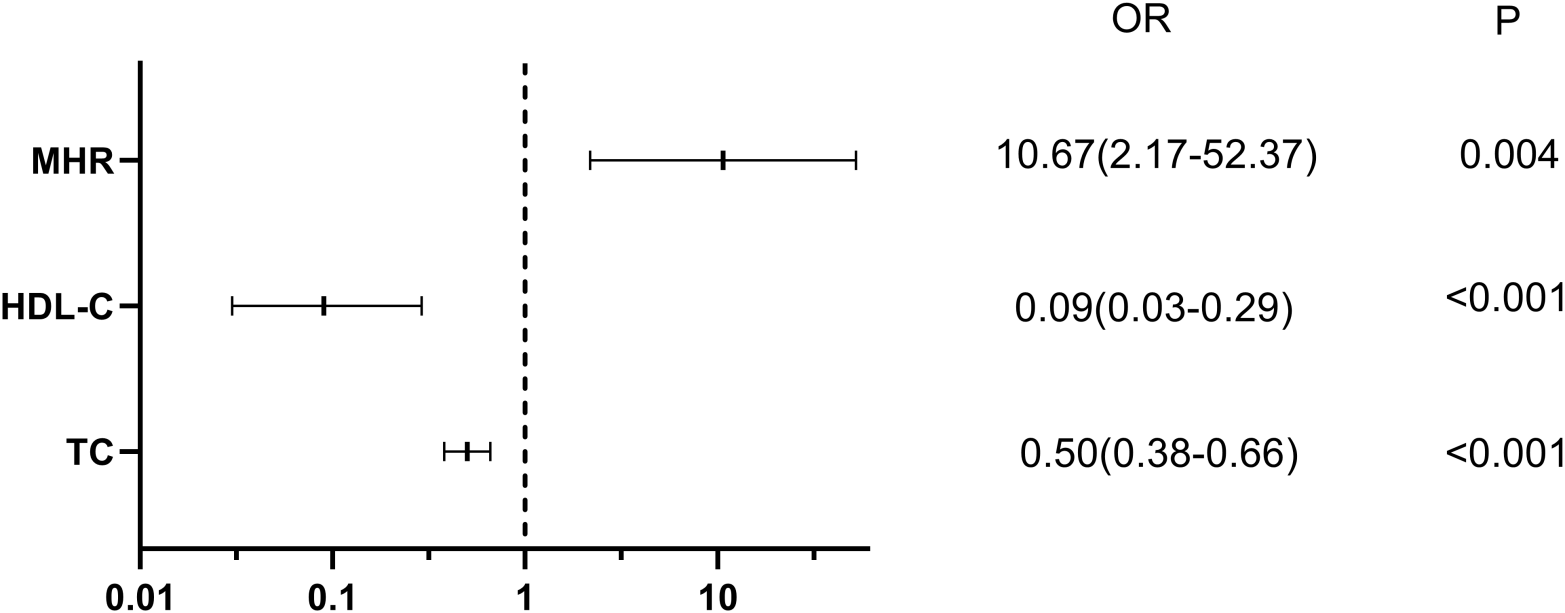
Forest plots of independent factors associated with AF in NAFLD.

The univariate logistic regression analysis showed that TC, HDL-C, MHR and NHR were significantly associated with the occurrence of AF in NAFLD patients. Further, multivariate logistic regression analysis identified that NHR was not a risk factor for AF in NAFLD patients, whereas MHR (OR, 10.67; 95% CI, 2.17-52.37; P = 0.004) was still independently associated with the presence of AF. TC (OR, 0.50; 95% CI, 0.38-0.66; P < 0.001) and HDL-C (OR, 0.09; 95% CI, 0.03-0.29; P < 0.001) were protective factors for AF (**Table 3**; **Figure 3**).

**Table 3:** Univariate and multivariate analyses of factors associated with NAFLD-AF.

	Non-adjusted		Model I		Model II	
	OR (95%CI)	P	OR (95%CI)	P	OR (95%CI)	P
TC (mmol/L)	0.62 (0.49-0.78)	<0.001	0.51 (0.39-0.67)	<0.001	0.50 (0.38-0.66)	<0.001
TG (mmol/L)	0.79 (0.60-1.04)	0.097				
HDL-C (mmol/L)	0.19 (0.07-0.50)	<0.001	0.10 (0.03-0.33)	<0.001	0.09 (0.03-0.29)	<0.001
LDL-C (mmol/L)	0.76 (0.56-1.04)	0.084				
MON (10^9^/L)	3.15 (0.81-12.24)	0.098				
LEM (10^9^/L)	1.04 (0.93-1.16)	0.521				
NEU (10^9^/L)	1.05 (0.95-1.15)	0.313				
MHR	10.29 (2.73-38.89)	<0.001	11.24 (2.34-53.89)	0.002	10.67 (2.17-52.37)	0.004
NHR	1.16 (1.03-1.32)	0.015	1.13 (0.99-1.30)	0.068		
NLR	1.11 (0.99-1.25)	0.079				

None, non-adjusted model. Model I was adjusted for age, sex and BMI. Model II was adjusted for age, sex, BMI, hypertension, diabetes mellitus, Coronary heart disease and smoking history.

### Association between MHR and the prevalent AF in the NAFLD

To understand the relationship between different MHR levels and AF in NAFLD patients, we divided the patients into tertiles according to MHR. As shown in **Table 4**, the risk of AF significantly rise as the MHR tertiles increased in the non-adjusted model, Model I and Model II (P for trend = 0.002, = 0.008, and = 0.013, respectively). Specifically, in non-adjusted model, a 2.37-fold increased risk of AF for patients with MHR values in MHR3 (OR 2.37; 95% CI, 1.34-4.16) was observed compared with the MHR1. When confounding factors were further adjusted in Model I and Model II, MHR still remained related to more than a twofold increased risk of AF. Meanwhile, in our analysis, no association was found between different levels of MHR and different types of AF (**Figure S1**).

**Table 4:** Univariate and multivariate logistic analyses of AF in tri-sectional MHR groups.

	Non-adjusted		Model I		Model II	
	OR (95%CI)	P	OR (95%CI)	P	OR (95%CI)	P
MHR1	Ref.		Ref.		Ref.	
MHR2	0.98 (0.56-1.72)	0.951	1.08 (0.59-1.97)	0.812	1.06 (0.57-1.97)	0.848
MHR3	2.37 (1.34-4.16)	0.003	2.33 (1.22-4.45)	0.010	2.24 (1.16-4.32)	0.016
P for trend	0.002		0.008		0.013	

None, non-adjusted model. Model I was adjusted for age, sex and BMI. Model II was adjusted for age, sex, BMI, hypertension, diabetes mellitus, Coronary heart disease and smoking history.

**Figure 4:**
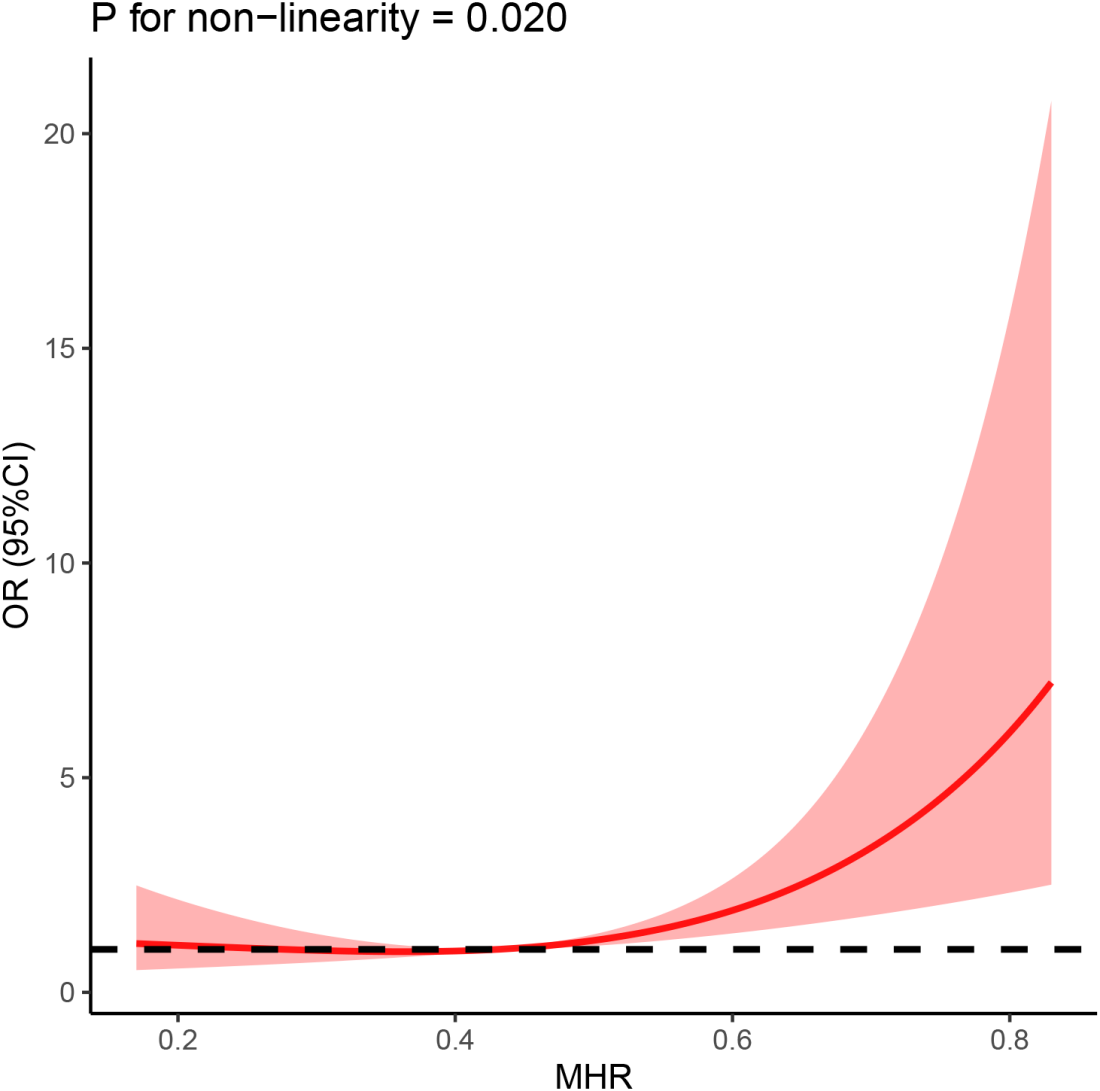
The association between MHR and AF was shown using restricted cubic splines (RCS), adjusting for age, sex, BMI, hypertension, diabetes mellitus, Coronary heart disease and smoking history.

Our study further employed the restricted cubic splines (RCS) to validate the correlation between MHR and the risk of prevalent AF, which was displayed in **Figure 4**. The RCS model showed a non-linear and J-shaped association between MHR and the prevalence of AF (P for non-linearity = 0.023), with a turning point of 0.44 by threshold effect analysis. A significant association was found after the turning point, but the association between MHR and risk of AF was not significant before the cut-off point.

### ROC analysis of MHR

**Figure 5:**
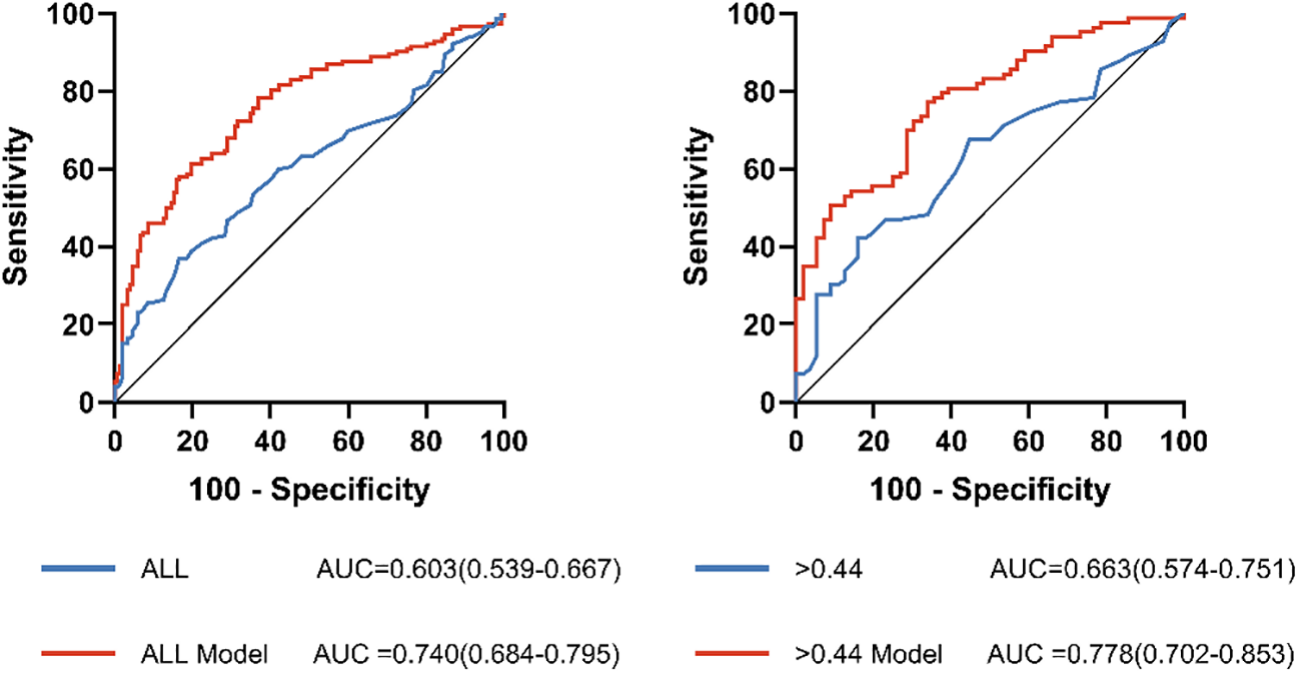
Receiver operating characteristic (ROC) curve analysis of the predictive power of MHR for AF. ALL Model, >0.44 Model: The new model integrates risk factors for AF (sex, age, BMI, hypertension, diabetes mellitus, Coronary heart disease and smoking history).

ROC analysis was performed to assess the usefulness of MHR in detecting the prevalent AF among NAFLD patients (**Table 5**; **Figure 5**). In the full range of MHR, the AUC of MHR alone was 0.603 (95% CI, 0.539-0.667; P = 0.002). When the rule-in threshold was 0.44, the AUC was 0.663 (95% CI, 0.574–0.751; P = 0.001), and the cut-off value of MHR was 0.655 (sensitivity, 42.7%; specificity, 85.2%). By adjusting for traditional risk factors (including age, gender, HT, DM, CHD, and smoking), MHR could improve the predicting ability for prevalent AF (0.663 vs. 0.778).

**Table 5:** ROC curve analyses of different levels of MHR.

Non-adjusted						
	AUC	95%CI	P	Sensitivity	Specificity	cut-off value
ALL	0.603	0.539-0.667	0.002	0.373	0.836	0.566
MHR ≤ 0.44	0.476	0.384-0.568	0.608	–	–	–
MHR>0.44	0.663	0.574-0.751	0.001	0.427	0.852	0.655
Model I						
ALL	0.740	0.684-0.795	<0.001	0.579	0.809	0.558
MHR>0.44	0.778	0.702-0.853	<0.001	0.771	0.661	0.559

None, non-adjusted model. Model I was adjusted for age, sex, BMI, hypertension, diabetes mellitus, Coronary heart disease and smoking history.

## Discussion

NAFLD is by far the most common metabolic disease in the world, placing a heavy burden on human health and health care systems, a burden that comes overwhelmingly from an increase in cardiovascular disease (15). AF is one of the most common arrhythmias worldwide, and it increases the risk of systemic stroke and heart failure (8). A growing number of studies have shown a strong association between NAFLD and AF, and the disease is receiving increasing attention as an emerging risk factor for AF (16). In a prospective cohort study, the authors found that NAFLD remained independently associated with the risk of AF (OR 1.88; 95% CI 1.03-3.45) after adjusting for multiple confounders (6). Meanwhile, in 2020, a meta-analysis of six studies including 614,673 individuals showed that patients with NAFLD had a significantly higher risk of AF than those without it (17). In summary, NAFLD is strongly associated with an increased risk of AF, but the potential intrinsic association between them is unclear. It is currently believed that systemic inflammation, insulin resistance, oxidative stress, and expansion of epicardial adipose tissue (EAT) may bridge the gap between NAFLD and AF (8).

In NAFLD, excessive accumulation of hepatic fat can lead to significant metabolic stress inflammation, enhancing the local inflammatory response and promoting progression from simple steatosis degeneration to nonalcoholic steatohepatitis (NASH) and fibrosis (18). Activation of hepatic immune signaling can lead to the release of pro-inflammatory cytokines and chemokines that enter the body circulation through the hepatic sinusoids and enhance the systemic inflammatory response (19, 20). Increasing evidence suggests that circulating markers of the systemic inflammatory response, including hs-CRP, TNF-a, IL-1, IL-6, and IL-18, are increased in patients with NAFLD and experimental models (21, 22). According to the aforementioned data, NAFLD can result in systemic inflammation that may have an impact on distant tissues like the heart.

In addition, the important role of chronic low-grade inflammation in the pathophysiology of arrhythmia has been increasingly recognized (23). A meta-analysis showed that high plasma levels of inflammatory markers such as IL-6 and CRP were associated with an increased risk of AF and the recurrence of AF after radiofrequency ablation or cardioversion (24). Elevated inflammatory mediators may lead to changes in the electrophysiology, structure, and function of the heart, leading to the occurrence of arrhythmia. Several studies have shown that pro-inflammatory cytokines affect the expression and function of calcium and potassium ion channels, causing the cardiomyocyte action potential and QTc interval to be prolonged and thus promoting the development of arrhythmias (25). Meanwhile, high levels of circulating CRP and IL-6 were positively correlated with atrial diameter (26). In addition, TNF-α has been reported to activate the fibrotic pathway (24).

Epicardial adipose tissue (EAT) may also be another important mediator in the association between NAFLD and AF. A cross-sectional study (27) found that epicardial fat thickness was independently associated with hepatic fibrosis severity. At the same time, indicators of cardiac morphology and function were also closely related to the severity of liver injury. EAT, as an ectopic adipose pool, is in close contact with cardiomyocytes and the epicardial membrane, exerting its paracrine function and affecting the structure and function of the heart. In a healthy state, EAT can secrete many mediators called “adipose metabolites”, including adiponectin, which play an anti-inflammatory, anti-fibrosis, and antioxidant role (28). However, in the case of systemic inflammation caused by metabolic disorders (such as NAFLD) and the accompanying accumulation of EAT, the secretion of adiponectin is decreased, while EAT releases a large number of inflammatory mediators (such as IL-6, IL-8, TNF-a, and leptin), aggravating the inflammatory environment and adversely affecting the myocardial structure and electrical remodeling (29, 30). The gradual accumulation of EAT leads to more extensive fat infiltration of the myocardium, a phenomenon closely associated with myocardial fibrosis, increased inflammation, and increased conduction block. An animal experiment conducted by Venteclef et al. (31) found that the human EAT secretion group could induce atrial fibrosis in rats, and the adipokine Activin A played an important role in promoting fibrosis. In conclusion, the paracrine effects of ectopic fat (such as EAT) around the heart in patients with NAFLD may lead to structural and electrophysiological changes in the heart, which may lead to arrhythmia.

Taken together, atrial electrophysiology and structure may be altered by NAFLD-triggered inflammatory responses, which may lead to increased susceptibility to AF. The activation of monocytes plays a crucial role in the whole process of chronic inflammation (32). Monocytes are a heterogeneous population that can be divided into three subpopulations with differences in function and phenotype, whereas monocytes of the CD14++/CD16+ (Mon2) phenotype are strong producers of cytokines after stimulation (e.g., TNF-α) (33). Suzuki et al. (34) previously reported an increased proportion of CD14++CD16+ intermediate monocyte subsets in AF subjects compared to healthy controls, and Fontes et al. (32) showed a higher circulating monocyte ratio associated with postoperative AF during cardiac surgery. Secondly, it was found that the enhanced migration activity of circulating monocytes and the increased number of atrial wall monocytes/macrophages may be the basis of LA remodeling and AF pathophysiology (35). Therefore, monocytes and various cytokines and differentiated macrophages accelerate cardiomyocyte inflammatory response and tissue remodeling, which increases the risk of AF.

On the other hand, monocytogenic cells, including phagocytes and Kupffer cells, are also key regulators of the progression and regression of liver fibrosis in non-alcoholic steatohepatitis (NASH) (36). Large amounts of experimental and clinical data have shown that monocyte infiltration increases in NASH model (37). Moreover, inhibiting the migration and recruitment of monocytes can alleviate liver inflammation and fibrosis (38). It is worth noting that the inflammatory process promoted by monocytes through interleukin-1β and TNF-α can be inhibited by increasing plasma HDL (39). In addition, HDL-C also exhibits an antioxidant response to monocytes by inhibiting their activation and proliferation (40).

Inflammation and oxidative stress, as major contributors to CVD, have been identified as risk factors for AF. Mediators of the inflammatory response can lead to electrical and structural remodeling of the atria, thereby increasing susceptibility to AF. As an important source of pro-inflammatory and pro-oxidative factors, monocytes play a key role in the initiation, continuation and recurrence of AF by activating the inflammatory cascade. The relationship between HDL-C and CVD has been extensively studied. Low HDL-C adversely affects the pathogenesis of AF by decreasing its anti-inflammatory and antioxidant effects, which is consistent with our results. In conclusion, monocytes combined with HDL-C could better predicted the risk of AF in NAFLD patients.

Based on the inflammatory profile of monocytes and the anti-inflammatory profile of HDL cholesterol, MHR has been proposed as a new indicator of inflammation (41–43). In the current study, MHR has emerged as a new and valuable biomarker for predicting cardiovascular disease, providing valid clinical information (44). In 2004, for the first time, increased MHR was found to be strongly associated with an elevated risk of cardiovascular events in patients with chronic kidney disease (44). Moreover, MHR is clinically important in predicting both early recurrence and late recurrence of AF after catheter ablation (11, 45, 46). Meanwhile, MHR was found to be significantly associated with the organism’s left atrial size, CRP, and BNP levels (46). All these results support the important role of MHR in the development of atrial fibrillation. NAFLD is gaining attention as an important independent risk factor for atrial fibrillation. Multiple cross-sectional studies (12, 47) have shown that MHR is closely associated with an increased risk of NAFLD and can be used for early monitoring of NAFLD. However, no study has investigated whether MHR predicts the risk of developing AF in the NAFLD population. Therefore, our study aimed to investigate this association.

Our findings indicate that MHR is closely related to the risk of developing AF. Interestingly, the association between the MHR index as a continuous measure and the AF hazard ratio in patients with NAFLD was J-shaped. Although this is relatively common (48), this has important clinical implications as it can highlight the threshold of the index. In this case, we believe that 0.44 May be the threshold of MHR index. When the MHR index is less than 0.44, it has no effect on the occurrence of atrial fibrillation; when the index is greater than 0.44, the risk of atrial fibrillation is significantly increased. We suggest that this result may be influenced by HDL-C. Low levels of HDL-C have been reported to lead to atrial myocyte abnormalities by reducing reverse cholesterol transport, and also indirectly to decreased anti-inflammatory and antioxidant effects (49). When the MHR index is too small, the harmful effects of higher monocyte levels can be balanced with the preventive effects of very high HDL-C. Resulting in low MHR does not affect the occurrence of atrial fibrillation. On the other hand, when the MHR index continues to rise, the deleterious effects of monocytes gradually emerge and represent higher levels of inflammation, which greatly increases the risk of AF. However, these results still need to be confirmed in prospective trials with large samples. In addition, ROC analysis showed that MHR had a high predictive power for the risk of developing AF. Therefore, MHR may become a new inflammatory indicator for predicting the risk of developing AF among NAFLD patients.

Our study has some limitations. First, this study is a retrospective cross-sectional study with a small single-center sample, which may have some impact on the results. Second, clinical confounders not included in this study may affect the accuracy of the results. Third, The diagnosis of NAFLD is made by ultrasonography. Ultrasonography is not the most sensitive method for the diagnosis of fatty liver, and there are some false positives. However, ultrasonography is simple, convenient and repeatable, so it is still the most effective method for clinical diagnosis of NAFLD. Finally, this study is a cross-sectional study, and its results can only indicate that MHR is associated with the risk of AF among patients with NAFLD but not its predictive value. A large number of large-scale, multicenter prospective studies are still needed in the future to further illustrate the predictive value of MHR for the development of AF in patients with NAFLD.

## Conclusion

In conclusion, our study found that MHR was significantly higher in patients with NAFLD combined with AF than in non-AF patients. MHR, as a simple and practical new inflammatory index, could be used to assess the risk of AF in the clinical management of NAFLD patients.

## Data availability statement

The original contributions presented in the study are included in the article/**Supplementary Material**. Further inquiries can be directed to the corresponding author.

## Ethics statement

The studies involving human participants were reviewed and approved by the Medical Ethics Committee of Shanxi Medical University. Written informed consent for participation was not required for this study in accordance with the national legislation and the institutional requirements.

## Author contributions

LW and BL: study design. LW and YZ: data analysis. LW and JZ: manuscript drafting. LW, YZ and BY: data collection. WZ, HF, ZR and BL: review and final approval. All authors contributed to the article and approved the submitted version.
