# Supplementary figures and images for: The monocyte-to-high-density lipoprotein ratio is associated with the occurrence of atrial fibrillation among NAFLD patients: A propensity-matched analysis
Source: Front Endocrinol (Lausanne). 2023 Mar 28;14:1127425. doi: 10.3389/fendo.2023.1127425 (PMC10086442; doi:10.3389/fendo.2023.1127425)

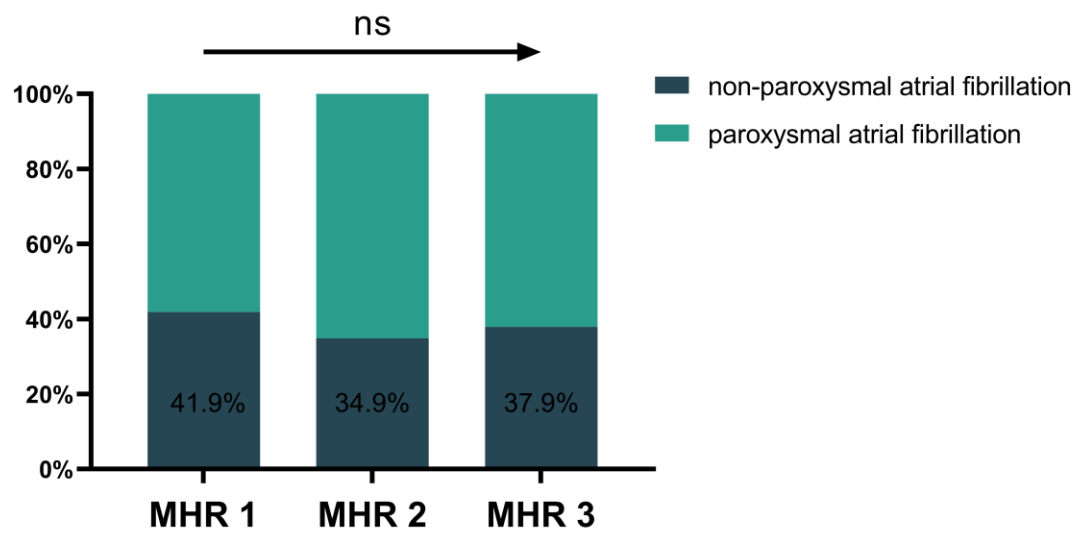

Fig.S1 The relationship between different levels of MHR and different types of AF.

Supplement: Supplementary file 1 [file Image_1.pdf]
